# A General Accelerated Degradation Model Based on the Wiener Process

**DOI:** 10.3390/ma9120981

**Published:** 2016-12-06

**Authors:** Le Liu, Xiaoyang Li, Fuqiang Sun, Ning Wang

**Affiliations:** 1 School of Reliability and Systems Engineering, Beihang University, Beijing 100191, China; liule@buaa.edu.cn (L.L.); leexy@buaa.edu.cn (X.L.); wnasd@buaa.edu.cn (N.W.); 2 Science and Technology on Reliability and Environmental Engineering Laboratory, Beijing 100191, China

**Keywords:** accelerated degradation testing, Wiener process, reliability, uncertainty, unit-to-unit variation

## Abstract

Accelerated degradation testing (ADT) is an efficient tool to conduct material service reliability and safety evaluations by analyzing performance degradation data. Traditional stochastic process models are mainly for linear or linearization degradation paths. However, those methods are not applicable for the situations where the degradation processes cannot be linearized. Hence, in this paper, a general ADT model based on the Wiener process is proposed to solve the problem for accelerated degradation data analysis. The general model can consider the unit-to-unit variation and temporal variation of the degradation process, and is suitable for both linear and nonlinear ADT analyses with single or multiple acceleration variables. The statistical inference is given to estimate the unknown parameters in both constant stress and step stress ADT. The simulation example and two real applications demonstrate that the proposed method can yield reliable lifetime evaluation results compared with the existing linear and time-scale transformation Wiener processes in both linear and nonlinear ADT analyses.

## 1. Introduction

Due to the highly competitive market, nowadays many products are requested to have long lifespans and high reliability. In order to quantify their lifetime and reliability characteristics, accelerated degradation testing (ADT) is conducted under harsh environmental conditions to accelerate the performance degradation and obtain sufficient data for reliability analysis in a short time with a limited budget [1,2,3]. Thus, it has been widely used in many engineering applications, e.g., batteries [4,5], light emitting diodes (LEDs) [6,7], etc.

The aim of statistical analysis of ADT data is to extrapolate product characteristics that are of interest at normal stress levels which, in general, comes from two directions. In the time direction, the deterministic degradation trend varying with time should be modeled at all accelerated levels for linear or nonlinear scenarios, i.e., the degradation model. In the stress direction, the relationship between acceleration variables and degradation-related parameters need to be established based on physical mechanisms or empirical experience, i.e., an acceleration model which will be used for the lifetime and reliability extrapolation at the normal stress level. In addition, the uncertainties from the temporal variation of the degradation process and unit-to-unit variation from the inherent heterogeneity of the tested products should be properly considered when analyzing ADT data. In the literature, two classes of models are widely used for degradation modeling, i.e., degradation path models [8,9] and stochastic process models [10,11,12], with examples for acceleration models including the Arrhenius model for temperature stress and the Eyring model for voltage stress [3,8,13]. With different combinations of degradation and acceleration models from these two directions, extensive work has been given to the statistical analysis of ADT data.

Since the stochastic process models can describe the temporal variation of the degradation process in a finite time interval, more attention has been given to them. In linear ADT analysis with a single acceleration variable, Park and Padgett [14] proposed two accelerated degradation models based on geometric Brownian motion and the Gamma process, and analyzed the constant stress ADT (CSADT) data of carbon-film resistors under temperature stress. Pan and Balakrishnan [15] proposed two linear modeling methods based on the Wiener and Gamma processes, and simulated one step stress ADT (SSADT) data with the Arrhenius model for model verification. Wang, et al. [16] used a linear Wiener process to model the degradation process for reliability evaluation of products with the integrated ADT and field information. In the cases with multiple acceleration variables, Liao and Elsayed [17] selected Brownian motion with a linear drift as the degradation model to infer the field reliability and applied it to LED CSADT data with electric current and ambient temperature.

In reality, the degradation processes of some products may experience nonlinear due to the inner deterioration mechanism of the product material, e.g., crack growth. In order to analyze this kind of ADT data, a time-scale transformation is generally used based on linear stochastic process models with a single acceleration variable. To our knowledge, Whitmore and Schenkelberg [11] were the first to accomplish this work in the area of ADT and proposed a time-scale transformation Wiener process model for the cable CSADT data analysis under temperature stress. A similar transformation is introduced in the Gamma process [10] and inverse Gaussian process models [12] for ADT modeling. In consideration of the unit-to-unit variation, Tang, et al. [18] incorporated a random variable into the acceleration model to capture the random effect and also used the time-scale transformation Wiener process model for nonlinear LED CSADT data analysis under electric current stress. However, the problem of using time-scale transformation is the implied assumption that the degradation processes can be linearized. It may not be suitable for nonlinear ADT analysis where the degradation paths cannot be linearized. In traditional degradation analysis, Wang, et al. [19,20] proposed a general Wiener model which utilizes two time-scale parameters to extend the above transformation and can solve the nonlinear degradation modeling to a greater extent. In our previous work [21], we introduced this model into ADT analysis, but without the consideration of unit-to-unit variation.

Based on the above research, it can be concluded that the existing methods partially solve the modeling problems of some ADT data with their applications in both the time and stress directions. However, more efforts are still needed, especially for nonlinear ADT modeling with multiple acceleration variables. Therefore, in this paper, a general ADT model is proposed to fill this gap based on the Wiener process. The general ADT model will consider the unit-to-unit variation and temporal variation of the degradation processes, and is suitable for both linear and nonlinear ADT analyses with single or multiple acceleration variables, simultaneously.

The rest of this paper is organized as follows: Section 2 introduces the general ADT model and derives its failure time distribution under the given stress level; Section 3 deals with the statistical inference of unknown parameters in the proposed model for both CSADT and SSADT; Section 4 uses one simulation example and two real applications to illustrate the superiority of the proposed method over other existing Wiener processes for both linear and nonlinear ADT analyses; and some concluding remarks are given in Section 5.

## 2. The General ADT Model

### 2.1. Models

The general ADT model based on the Wiener process is given by:

(1) $$M_{0}:X\left(t\right)=\mu \left(S;\eta \right)\Lambda \left(t;\theta \right)+\sigma B\left(\tau \left(t;\gamma \right)\right),$$

where *X*(*t*) is the performance degradation value of product at time *t*, *σ* denotes the diffusion coefficient, which is assumed to be constant, *B*(.) is the standard Brownian motion to describe the temporal variation of the degradation process, Λ(.) and *τ*(.) are the monotonous functions with time, *θ* and *γ* are the time-scale parameters to present the linear or nonlinear modeling. Without loss of generality, the initial degradation value is set to be zero. If not, *X*(*t*) = *X*(*t*) − *X*(0) will be used.

For the drift coefficient *μ*(***S***;***η***), it is assumed to be dependent with the acceleration variable ***S*** as the acceleration model. The acceleration model for both single and multiple acceleration variables is denoted as:

(2) $$\mu \left(S;\eta \right)=\eta_{0}\prod_{v=1}^{p}{\left[\phi \left(s_{v}\right)\right]}^{\eta_{v}}.$$

If there are *p* acceleration variables, then ***S*** = [*s*_1_, *s*_2_, …, *s_p_*] and ***η*** = [*η*_0_, *η*_1_, …, *η_p_*], where *s_v_* is the *v*th acceleration stress type and *η_v_* is the *v*th constant coefficient. While *φ*(.) is the continuous function of acceleration variables *s_i_*, *i* = 1, 2, …, *p*. For instance, if *φ*(*s*) = exp(1/*s*), Equation (2) presents the Arrhenius relationship (exponential type); while if *φ*(*s*) = *s*, Equation (2) presents the Eyring relationship (power rule type).

Considering the unit-to-unit variation due to the inherent heterogeneity among the tested products, the drift coefficients *μ*(***S***;***η***) are varied from product to product. Hence, we consider it as a random variable to present this kind of variation. Similar methods can be found in Peng and Tseng [22], Wang [23], and Si, et al. [24]. Here, for simplicity, the coefficient *η*_0_ in Equation (2) is assumed to follow a normal distribution with mean value *a* and variance *b*, i.e., *η*_0_ ~ *N*(*a*, *b*). The parameter values should be such that Pr(*μ*(***S***;***η***) < 0) is nearly zero to avoid negative values existing in the drift coefficient of Equation (1). Thus, if such a random effect is not considered (*b* = 0), Equation (2) will become the traditional acceleration model in Park and Padgett [25].

The attraction of Equation (1) is that it can cover two commonly used Wiener processes in the ADT field as its limiting cases, which are:

- **Case 1.** If Λ(*t*;*θ*) = *t* and *τ*(*t*;*γ*) = *t*, Equation (1) is simplified to the traditional linear Wiener process, which is widely used for ADT analysis, see [16,25,26,27] and so on:

(3) $$M_{1}:X\left(t\right)=\mu \left(S;\eta \right)t+\sigma B\left(t\right).$$

- **Case 2.** If Λ(*t*;*θ*) = *τ*(*t*;*γ*), Equation (1) reduces to a time-scale transformation Wiener process as in [11,18]:

(4) $$M_{2}:X\left(t\right)=\mu \left(S;\eta \right)\tau \left(t;\gamma \right)+\sigma B\left(\tau \left(t;\gamma \right)\right).$$

Herein, if the time-scale transformation *z* = *τ*(*t*;*γ*) is used in Equation (4), then Case 2 will become a similar form of Case 1 as *Y*(*z*) = *μ*(***S***;***η***)*z* + *σB*(*z*).

Furthermore, if the acceleration variables in Equation (2) are set to be at normal stress levels where *μ*(***S***;***η***) ~ *N*(*μ*_0_, $\sigma_{0}^{2}$), Equation (1) and its limiting cases (i.e., Equations (3) and (4)) are the degradation models used in traditional degradation analysis [19,20,28].

As described above, the general ADT model in Equation (1) can present the uncertainties from the unit-to-unit variation (*b* ≠ 0) and temporal variation of the degradation process, and can be used in the situations with single (*p* = 1) and multiple (*p* > 1) acceleration variables for linear and nonlinear degradation processes. For clarity, Equation (1) is named *M*_0_, while Equations (3) and (4) are *M*_1_ and *M*_2_. Since model *M*_1_ and *M*_2_ are widely used in the ADT field, in this paper, we concentrate on the comparison of *M*_1_ and *M*_2_ with *M*_0_ to verify the effectiveness of the proposed model for both linear and nonlinear ADT analyses.

For the evaluation of lifetime and reliability, the probability distribution function (PDF) of the failure time should be given, which will be derived in the following section.

### 2.2. Derivation of the Failure Time Distribution under the Given Stress Level

Although ADT is implemented at accelerated stress levels, the lifetime and reliability evaluation are conducted at the given normal stress level ***S***_0_ = [$s_{1}^{\left(0\right)}$, $s_{2}^{\left(0\right)}$,⋯, $s_{p}^{\left(0\right)}$], where $s_{v}^{\left(0\right)}$ is the *v*th acceleration stress type under normal level. Thus, the drift coefficient *μ*(***S***_0_;***η***) follows a normal distribution. Therefore, we simplify the notation of *μ*(***S***_0_;***η***) to *μ* in the derivation of the failure time distribution. From Equation (2), it is known that *μ* ~ *N*(*μ*_0_, $\sigma_{0}^{2}$), where $\mu_{0}=a\cdot \prod_{v=1}^{p}{\left[\phi (s_{v}^{\left(0\right)})\right]}^{\eta_{v}}$ and $\sigma_{0}^{2}=b\cdot \prod_{v=1}^{p}{\left[\phi (s_{v}^{\left(0\right)})\right]}^{2\eta_{v}}$.

In general, the failure time is defined as the time when degradation path *X*(*t*) first exceeds the failure threshold *ω*, i.e., the first passage time (FPT):

(5) T=inf{t:X(t)≥w}.

Let a time transformation *z* = *τ*(*t*;*γ*); thus *t* = *τ*^−1^(*z*;*γ*). We define *ρ*(*z*;*θ*) = Λ(*τ*^−1^(*z*;*γ*);*θ*). So, Equation (1) becomes [20]:

(6) Y(z)=μρ(z;θ)+σB(z),

and its drift coefficient is:

(7) $$\kappa \left(z;\theta \right)=\mu \frac{d\rho \left(z;\theta \right)}{dz}.$$

For simplicity, let *h*(*z*;*θ*) = *dρ*(*z*;*θ*)/*dz*. Under some mild assumptions, the PDF of the FPT for the new degradation process *Y*(*z*) is (see Theorem 2 in Si, et al. [24]):

(8) $$p_{Z}\left(z|\mu \right)=\frac{1}{\sqrt{2\pi z}}\left(\frac{\omega -\mu \rho \left(z;\theta \right)}{\sigma z}+\frac{\kappa \left(z;\theta \right)}{\sigma }\right)\cdot \exp\left(-\frac{{\left(\omega -\mu \rho \left(z;\theta \right)\right)}^{2}}{2\sigma^{2}z}\right).$$

In consideration of the random effect due to unit-to-unit variation, the uncertainty of *μ* should be included in the PDF of the FPT. We compute the result by the law of total probability, i.e.,:

(9) $$p_{Z}\left(z\right)=\int p_{Z}\left(z|\mu \right)p\left(\mu \right)d\mu .$$

In order to explicitly obtain the formula of Equation (9), Theorem 3 of Si, et al. [24] is introduced and modified accordingly; that is:

**Theorem** **1.** *If μ ~ N(μ_0_, $\sigma_{0}^{2}$), and ω, A, B, C*∈ ***R**, then*:

(10) $$E_{\mu }\left[\left(\omega -A\mu \right)\cdot \exp\left(-\frac{{\left(\omega -B\mu \right)}^{2}}{2C}\right)\right]=\sqrt{\frac{C}{B^{2}\sigma_{0}^{2}+C}}\cdot \left(\omega -A\frac{B\sigma_{0}^{2}\omega +\mu_{0}C}{B^{2}\sigma_{0}^{2}+C}\right)\cdot \exp\left(-\frac{{\left(\omega -B\mu_{0}\right)}^{2}}{2\left(B^{2}\sigma_{0}^{2}+C\right)}\right).$$

Therefore, we substitute Equations (8) into (9) according to Equation (10). The result is:

(11) $$p_{Z}\left(z\right)=\frac{1}{z\sqrt{2\pi Q}}\exp\left(-\frac{{\left(\omega -\rho \left(z;\theta \right)\mu_{0}\right)}^{2}}{2Q}\right)\cdot \left(\omega -\left(\rho \left(z;\theta \right)-h\left(z;\theta \right)z\right)\frac{\rho \left(z;\theta \right)\sigma_{0}^{2}\omega +\mu_{0}\sigma^{2}z}{Q}\right),$$

where *Q* = *ρ*^2^(*z*;*θ*)$\sigma_{0}^{2}$ + *σ*^2^*z*.

Hence, the PDF of the FPT for the general model can be obtained by the reverse process of the time transformation *z* = *τ*(*t*;*γ*) through Equation (11); that is:

(12) $$p_{T}\left(t\right)=\frac{1}{\tau \left(t;\gamma \right)\sqrt{2\pi Q}}\exp\left(-\frac{{\left(\omega -\Lambda \left(t;\theta \right)\mu_{0}\right)}^{2}}{2Q}\right)\cdot \left(\omega -G\frac{\Lambda \left(t;\theta \right)\sigma_{0}^{2}\omega +\mu_{0}\sigma^{2}\tau \left(t;\gamma \right)}{Q}\right)\frac{d\tau \left(t;\gamma \right)}{dt},$$

where *Q* = Λ^2^(*t*;*θ*)$\sigma_{0}^{2}$+ *σ*^2^*τ*(*t*;*γ*); *G* = Λ(*t*;*θ*) − *h*(*τ*(*t*;*γ*);*θ*)*τ*(*t*;*γ*).

Supposing that Λ(*t*;*θ*) = *t^θ^* and *τ*(*t*;*γ*) = *t^γ^*, Equation (12) becomes:

(13) $$p_{T}\left(t\right)=\frac{\gamma }{t\sqrt{2\pi \left(\sigma_{0}^{2}t^{2\theta }+\sigma^{2}t^{\gamma }\right)}}\exp\left(-\frac{{\left(\omega -\mu_{0}t^{\theta }\right)}^{2}}{2\left(\sigma_{0}^{2}t^{2\theta }+\sigma^{2}t^{\gamma }\right)}\right)\cdot \left(\omega -\frac{\left(\gamma -\theta \right)t^{\theta }\left(\omega \sigma_{0}^{2}t^{\theta }+\mu_{0}\sigma^{2}t^{\gamma }\right)}{\gamma \left(\sigma_{0}^{2}t^{2\theta }+\sigma^{2}t^{\gamma }\right)}\right).$$

Herein, the relationship ∫*p_T_*(*t*)*dt* = 1 should be satisfied. Therefore, the PDF and cumulative distribution function (CDF) of the FPT for model *M*_0_ are modified as:

(14) $$\begin{array}{l} f_{T}\left(t\right)≅p_{T}\left(t\right)/\int_{0}^{+\infty }p_{U}\left(u\right)du,\\ F_{T}\left(t\right)≅\int_{0}^{t}p_{U}\left(u\right)du/\int_{0}^{+\infty }p_{U}\left(u\right)du. \end{array}$$

For *M*_1_, i.e., Λ(*t*;*θ*) = *t* and *τ*(*t*;*γ*) = *t*, the PDF of the FPT for *M*_1_ is known as an inverse Gaussian distribution [29]. Considering the random effect, the PDF and CDF of the FPT are [22,28]:

(15) $$\begin{array}{l} f_{T}\left(t\right)=\frac{\omega }{\sqrt{2\pi t^{3}\left(\sigma_{0}^{2}t+\sigma^{2}\right)}}\exp\left(-\frac{{\left(\omega -\mu_{0}t\right)}^{2}}{2t\left(\sigma_{0}^{2}t+\sigma^{2}\right)}\right),\\ F_{T}\left(t\right)=\Phi \left(\frac{\mu_{0}t-\omega }{\sqrt{\sigma_{0}^{2}t^{2}+\sigma^{2}t}}\right)+\exp\left(\frac{2\mu_{0}\omega }{\sigma^{2}}+\frac{2\sigma_{0}^{2}\omega^{2}}{\sigma^{4}}\right)\cdot \Phi \left(-\frac{2\sigma_{0}^{2}\omega t+\sigma^{2}\left(\mu_{0}t+\omega \right)}{\sigma^{2}\sqrt{\sigma_{0}^{2}t^{2}+\sigma^{2}t}}\right). \end{array}$$

Obviously, Equation (15) is a limiting case of Equation (14) if we substitute *θ* = *γ* = 1 into Equation (13), where ∫*p_T_*(*t*)*dt* = 1.

For *M*_2_, i.e., Λ(*t*;*θ*) = *τ*(*t*;*γ*), the PDF of the FPT for *M*_2_ is in accordance with Equation (15) through a time-scale transformation by replacing t into Λ(*t*;*θ*) or *τ*(*t*;*γ*) [18,30], which is also a limiting case of Equation (14).

In the following section, the problems of parameter estimation will be addressed for CSADT and SSADT. After that, the PDF and CDF of the FPT at normal stress levels will be computed through Equation (14) for the lifetime and reliability evaluation of long lifespan products.

## 3. Statistical Inference

**Figure 1:**
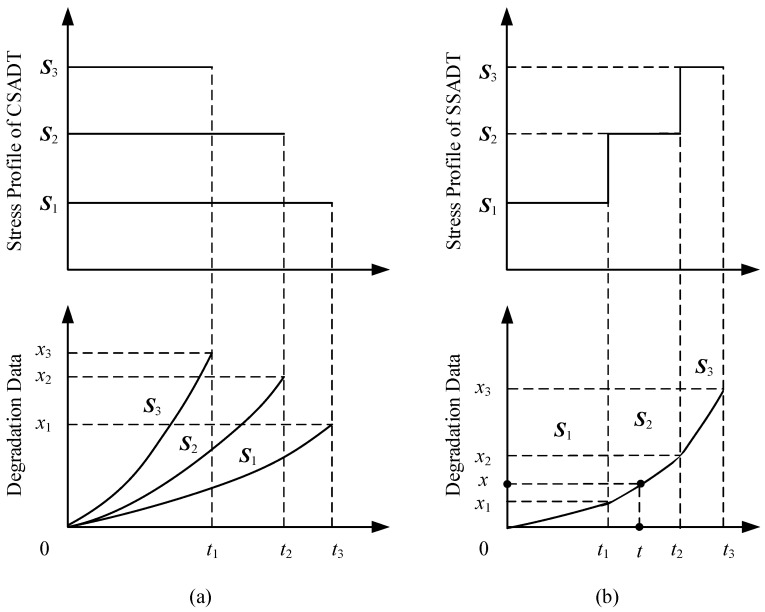
The schematic of (**a**) CSADT and (**b**) SSADT under three stress levels.

With different loading profiles of accelerated stresses, CSADT and SSADT are widely used in engineering applications. Figure 1a,b show the schematic of these two types of ADT under three stress levels. In the left, samples are separated into different groups and tested under different constant stress levels, i.e., ***S***_1_, ***S***_2_ and ***S***_3_, while, on the right, all samples are in the same group and tested from the lowest stress level to the highest in a step-by-step manner, i.e., ***S***_1_ → ***S***_2_ → ***S***_3_. Thus, the advantage of SSADT is that it can save the number of samples with a shorter test time [31].

Herein, we provide the method for estimating the unknown parameters in CSADT. Given in Section 2, the unknown parameter vector is *Ω* = [*θ*, *γ*, *σ*, *a*, *b*, *η_v_*], *v* = 1, 2, …, *p*. The analytic expressions of those parameters are hard to obtain directly. Hence, a two-stage maximum likelihood estimation (MLE) method is proposed to address this issue. In the first stage, the parameters related to the degradation process model are estimated, i.e., *Ω*_1_ = [*θ*, *γ*, *σ*] in Equation (1). In the second stage, the rest of the parameters related to the acceleration model, i.e., *Ω*_2_ = [*a*, *b*, *η_v_*] in Equation (2), are given accordingly.

### 3.1. Estimation of Ω_1_ for CSADT

The observed CSADT data *x_ijk_* is the *k*th degradation value of unit *j* under the *i*th stress level and *t_ijk_* is the corresponding measurement time, *i* = 1, 2, …, *K*, *j* = 1, 2, …, *n_i_*, *k* = 1, 2, …, *m_ij_*, where *K* is the number of stress levels, *n_i_* is the number of test samples under the *i*th stress level, and *m_ij_* is the number of measurements for unit *j* under the *i*th stress level. Let ***X****_ij_* = (*x_ij_*_1_, *x_ij_*_2_, …, *x_ijm__ij_*)^′^ and ***t****_ij_* = (Λ(*t_ij_*_1_;*θ*), Λ(*t_ij_*_2_;*θ*), …, Λ(*t_ijm__ij_*;*θ*))^′^. According to the properties of the Wiener process, the degradation value ***X****_ij_* follows a multivariate normal distribution:

(16) $$X_{ij}\;\sim \;N\left(\mu_{ij}t_{ij},\sigma^{2}Q_{ij}\right),$$

where *μ_ij_* is the drift coefficient of unit *j* under the *i*th stress level:

$$Q_{ij}=\left[\begin{array}{cccc} \tau \left(t_{ij1};\gamma \right) & \tau \left(t_{ij1};\gamma \right) & \cdots & \tau \left(t_{ij1};\gamma \right)\\ \tau \left(t_{ij1};\gamma \right) & \tau \left(t_{ij2};\gamma \right) & \cdots & \tau \left(t_{ij2};\gamma \right)\\ \vdots & \vdots & \ddots & \vdots \\ \tau \left(t_{ij1};\gamma \right) & \tau \left(t_{ij2};\gamma \right) & \cdots & \tau \left(t_{ijm_{ij}};\gamma \right) \end{array}\right].$$

Let $\mu =\left(\mu_{11},\cdots ,\mu_{1n_{1}},\cdots ,\mu_{Kn_{K}}\right)$. The likelihood function of the CSADT data can be easily obtained by Equation (16) and the logarithm function is:

(17) $$l\left(\mu ,\sigma ,\theta ,\gamma |X\right)=-\frac{ln\left(2\pi \right)}{2}\sum_{i=1}^{K}\sum_{j=1}^{n_{i}}m_{ij}-\frac{1}{2}\sum_{i=1}^{K}\sum_{j=1}^{n_{i}}ln\left|\sigma^{2}Q_{ij}\right|-\frac{1}{2}\sum_{i=1}^{K}\sum_{j=1}^{n_{i}}{\left(X_{ij}-\mu_{ij}t_{ij}\right)}^{\prime }\sigma^{-2}Q_{ij}^{-1}\left(X_{ij}-\mu_{ij}t_{ij}\right).$$

The first partial derivative of Equation (17) to $\mu_{ij}$ and $\sigma^{2}$ are:

(18) $$\frac{\partial l\left(\mu ,\sigma ,\theta ,\gamma |X\right)}{\partial \mu_{ij}}=X_{ij}^{\prime }\sigma^{-2}Q_{ij}^{-1}t_{ij}-\mu_{ij}t_{ij}^{\prime }\sigma^{-2}Q_{ij}^{-1}t_{ij},$$

(19) $$\frac{\partial l\left(\mu ,\sigma ,\theta ,\gamma |X\right)}{\partial \sigma^{2}}=-\frac{1}{2\sigma^{2}}\sum_{i=1}^{K}\sum_{j=1}^{n_{i}}m_{ij}+\frac{1}{2\sigma^{4}}\sum_{i=1}^{K}\sum_{j=1}^{n_{i}}{\left(X_{ij}-\mu_{ij}t_{ij}\right)}^{\prime }Q_{ij}^{-1}\left(X_{ij}-\mu_{ij}t_{ij}\right).$$

The maximum value of the log-likelihood function in Equation (17) is obtained when Equations (18) and (19) are equal to zero. Thus, the estimates of ${\hat{\mu }}_{ij}$ and ${\hat{\sigma }}^{2}$ relying on *θ* and *γ* are:

(20) $${\hat{\mu }}_{ij}=\frac{X_{ij}^{\prime }Q_{ij}^{-1}t_{ij}}{t_{ij}^{\prime }Q_{ij}^{-1}t_{ij}},$$

(21) $${\hat{\sigma }}^{2}=\frac{\sum_{i=1}^{K}\sum_{j=1}^{n_{i}}{\left(X_{ij}-{\hat{\mu }}_{ij}t_{ij}\right)}^{\prime }Q_{ij}^{-1}\left(X_{ij}-{\hat{\mu }}_{ij}t_{ij}\right)}{\sum_{i=1}^{K}\sum_{j=1}^{n_{i}}m_{ij}}.$$

Substituting Equations (20) and (21) into (17), the log-likelihood function is only a function of *θ* and *γ*, i.e.,:

(22) $$l\left(\theta ,\gamma |X\right)=-\frac{ln\left(2\pi \right)}{2}\sum_{i=1}^{K}\sum_{j=1}^{n_{i}}m_{ij}-\frac{1}{2}\sum_{i=1}^{K}\sum_{j=1}^{n_{i}}ln\left|{\hat{\sigma }}^{2}Q_{ij}\right|-\frac{1}{2}\sum_{i=1}^{K}\sum_{j=1}^{n_{i}}{\left(X_{ij}-{\hat{\mu }}_{ij}t_{ij}\right)}^{\prime }{\hat{\sigma }}^{-2}Q_{ij}^{-1}\left(X_{ij}-{\hat{\mu }}_{ij}t_{ij}\right).$$

Thus, $\hat{\theta }$ and $\hat{\gamma }$ can be obtained by the two-dimensional search for the maximum value of Equation (22) [32]. Then, the estimation of ${\hat{\mu }}_{ij}$ and ${\hat{\sigma }}^{2}$ can be easily computed by substituting $\hat{\theta }$ and $\hat{\gamma }$ into Equations (20) and (21).

### 3.2. Estimation of Ω_2_ for CSADT

The estimate of *Ω*_2_ is related to the acceleration model in Equation (2). From Section 3.1, the estimates of the drift coefficients $\mu_{ij}$ for unit *j* under the *i*th stress level are given and the corresponding stresses are $s_{v}^{\left(i\right)}$, *i* = 1, 2, …, *K*, *j* = 1, 2, …, *n_i_*. With the consideration of unit-to-unit variation in Equation (2), the relationship among them is denoted as:

(23) $$\mu_{ij}\sim N\left(a\cdot \prod_{v=1}^{p}{\left[\varphi \left(s_{v}^{\left(i\right)}\right)\right]}^{\eta_{v}},b\cdot \prod_{v=1}^{p}{\left[\varphi \left(s_{v}^{\left(i\right)}\right)\right]}^{2\eta_{v}}\right),$$

where $\mu_{ij}$ will be replaced by its estimate ${\hat{\mu }}_{ij}$ from Equation (20).

For simplicity, let $\left(s_{v}^{\left(i\right)}|\eta_{v}\right)=\prod_{v=1}^{p}{\left[\varphi \left(s_{v}^{\left(i\right)}\right)\right]}^{\eta_{v}}$. Thus, the log-likelihood function for *Ω*_2_ is:

(24) $$l^{\prime }\left(a,b,\eta_{v}|\hat{\mu }\right)=-\frac{ln\left(2\pi \right)}{2}\sum_{i=1}^{K}n_{i}-\frac{1}{2}\sum_{i=1}^{K}\sum_{j=1}^{n_{i}}\left(lnb+2ln\left(s_{v}^{\left(i\right)}|\eta_{v}\right)\right)-\frac{1}{2}\sum_{i=1}^{K}\sum_{j=1}^{n_{i}}\frac{{\left({\hat{\mu }}_{ij}-a\left(s_{v}^{\left(i\right)}|\eta_{v}\right)\right)}^{2}}{b{\left(s_{v}^{\left(i\right)}|\eta_{v}\right)}^{2}}.$$

Similar to the estimation procedure of *Ω*_1_, we compute the estimation of $\hat{a}$ and $\hat{b}$ relying upon $\left(s_{v}^{\left(i\right)}|\eta_{v}\right)$, which are:

(25) $$\hat{a}=\frac{1}{\sum_{i=1}^{K}n_{i}}\sum_{i=1}^{K}\sum_{j=1}^{n_{i}}\frac{{\hat{\mu }}_{ij}}{\left(s_{v}^{\left(i\right)}|\eta_{v}\right)},\;\hat{b}=\frac{1}{\sum_{i=1}^{K}n_{i}}{\sum_{i=1}^{K}\sum_{j=1}^{n_{i}}\left(\frac{{\hat{\mu }}_{ij}}{\left(s_{v}^{\left(i\right)}|\eta_{v}\right)}-\hat{a}\right)}^{2}.$$

Then, substituting Equations (25) into (24), the ${\hat{\eta }}_{v}$ can be obtained by a *p* dimensional search for the maximum value of Equation (24). After that, the estimates of $\hat{a}$ and $\hat{b}$ can be given by Equation (25), accordingly.

### 3.3. Estimation of Ω_1_ and Ω_2_ for SSADT

The estimates of unknown parameters in SSADT are slightly different from that in CSADT. If we divide SSADT data by the number of the stress levels *K* and set the initial values at each stress level to be zero, the SSADT data can be transformed into CSADT data. For instance, in Figure 1b, the degradation values from 0 to *x*_1_ during the time interval $[0,t_{1}]$ is obviously a subset of CSADT data. For the time intervals $(t_{1},t_{2}]$ and $(t_{2},t_{3}]$, a transformation will be given since the initial values of both time and degradation value are not zero, as they are in CSADT.

Keeping this in mind, we assume that $t\in (t_{1},t_{2}]$ and its corresponding degradation value is $x\in (x_{1},x_{2}]$. According to the properties of the Wiener process, the degradation value *x* satisfies that *x* − *x*_1_ ~ *N*(*μ*_2_(Λ(*t*;*θ*) − Λ(*t*_1_;*θ*)), *σ*^2^(*τ*(*t*;*γ*) − *τ*(*t*_1_;*γ*))) where *μ*_2_ is the drift coefficient when $t\in (t_{1},t_{2}]$. Given that *x*^′^ = *x* − *x*_1_, Λ^′^(*t*;*θ*) = Λ(*t*;*θ*) − Λ(*t*_1_;*θ*) and *τ*^′^(*t*;γ) = *τ*(*t*;*γ*) − *τ*(*t*_1_;*γ*). The SSADT data in the time interval $(t_{1},t_{2}]$ can be interpreted as a subset of CSADT data where the transferred degradation value is from 0 to *x*^′^ during the time interval $[0,t^{*}]$, where $t^{*}$ is Λ^′^(*t*;*θ*) or *τ*^′^(*t*;*γ*) accordingly. This procedure is the same for the data in the time interval (*t*_2_, *t*_3_].

Following this idea, let the observed SSADT data be *x_ijk_*, which is the *k*th degradation value of unit *j* at the stress level *i* and *t_ijk_* is the corresponding measurement time, *i* = 1, 2, …, *K*, *j* = 1, 2, …, *n*, *k* = 1, 2, …, *m_ij_*. The transformations are given in Equations (26)–(28):

(26) $$X_{ij}=\left[\begin{array}{c} x_{ij1}\\ x_{ij2}\\ \vdots \\ x_{ijm_{ij}} \end{array}\right]-x_{\left(i-1\right)jm_{\left(i-1\right)j}}\cdot I_{m_{ij}\times 1},$$

(27) $$t_{ij}=\left[\begin{array}{c} \Lambda \left(t_{ij1};\theta \right)\\ \Lambda \left(t_{ij2};\theta \right)\\ \vdots \\ \Lambda \left(t_{ijm_{ij}};\theta \right) \end{array}\right]-\Lambda \left(t_{\left(i-1\right)jm_{\left(i-1\right)j}};\theta \right)\cdot I_{m_{ij}\times 1},$$

(28) $$Q_{ij}=\left[\begin{array}{cccc} \tau \left(t_{ij1};\gamma \right) & \tau \left(t_{ij1};\gamma \right) & \cdots & \tau \left(t_{ij1};\gamma \right)\\ \tau \left(t_{ij1};\gamma \right) & \tau \left(t_{ij2};\gamma \right) & \cdots & \tau \left(t_{ij2};\gamma \right)\\ \vdots & \vdots & \ddots & \vdots \\ \tau \left(t_{ij1};\gamma \right) & \tau \left(t_{ij2};\gamma \right) & \cdots & \tau \left(t_{ijm_{ij}};\gamma \right) \end{array}\right]-\tau \left(t_{\left(i-1\right)jm_{\left(i-1\right)j}};\gamma \right)\cdot I_{m_{ij}\times m_{ij}},$$

where *I_m_*_×*n*_ is the *m* × *n* all ones matrix; *t*_0*jk*_ = 0 and *x*_0*jk*_ = 0.

Then, the unknown parameters in SSADT can be given by the proposed two-stage MLE method for CSADT, as shown in Section 3.1 and Section 3.2.

## 4. Case Study

In this section, one simulation example and two real cases are used to demonstrate the validity and superiority of the proposed models and methods over other existing forms of the Wiener processes on linear and nonlinear ADT analyses.

The literature has shown that the expectation of the degradation path can be formulated by an exponential function [33]. Therefore, the forms Λ(*t*;*θ*) = *t^θ^* and *τ*(*t*;*γ*) = *t^γ^* will be used in this paper. In order to compare the *s*-fits of model *M*_0_ with *M*_1_ and *M*_2_, the Akaike information criterion (*AIC*) is introduced:

(29) $$AIC=-2l_{max}+2n_{p},$$

where *l_max_* is the maximum value of the log-likelihood function in Equation (17), and *n_p_* is the number of unknown parameters. The lower the *AIC* value is, the better the model fits.

According to Burnham and Anderson [34], it is imperative to rescale *AIC* to:

(30) $$\Delta_{i}=AIC_{i}-AIC_{\min},$$

where *AIC_min_* is the minimum of *AIC_i_* values. Then, Δ = 0 for the best model, Δ ≤ 2 for models having substantial support, 4 ≤ Δ ≤ 7 for models having considerably less support and Δ > 10 for models having essentially no support compared to the best models.

In addition, for illustration purpose of the fitting results, a quantile-quantile (Q-Q) plot is used to graphically present the goodness of fit of each model on ADT data with the following standard normal distribution:

(31) $$\begin{array}{cc} \frac{x_{ijk}-{\hat{\mu }}_{ij}\Lambda \left(t_{ijk};\theta \right)}{\sigma \sqrt{\tau \left(t_{ijk};\gamma \right)}}\sim N\left(0,1\right) & i=1,2,\cdots ,K,j=1,2,\cdots ,n_{i},k=1,2,\cdots ,m_{ij} \end{array},$$

where the plot is approximately linear if the fitting is satisfactory.

### 4.1. Simulation Example

SSADT data is simulated under three temperature stress levels, *T* = 60 °C, 100 °C, and 120 °C, and the normal temperature of 25 °C. The observation time interval is one hundred hours, while the number of observations under three stress levels are 15, 10 and 5, respectively. Hence, the total test time is 3000 h. In order to evaluate the influence of the sample size on parameter estimation and the reliability evaluation, we conduct three simulation analyses under the sample size *n* = 5, 10 and 30, respectively. The failure threshold *ω* is 100. Without a loss of generality, the parameters are set to be *θ* = 1.5, *γ* = 0.4, *σ*^2^ = 0.01, *a* = 20, *b* = 5, and *η*_1_ = −1500. The Arrhenius model is selected as the acceleration model, which is:

(32) $$\mu \left(T;\eta_{0},\eta_{1}\right)=\eta_{0}\exp\left(\frac{\eta_{1}}{273.15+T}\right).$$

First, we check the normal assumption of *μ*_0_ at the normal stress level. Given by Equation (32) and the parameter settings, Pr(*μ*_0_ < 0) = 1.8720 × 10^−19^, which is approximately near zero. Thus, the non-negative assumption can be satisfied to compute the PDF of the FPT. In the following, both the model comparison and sensitivity analysis are conducted with the abovementioned parameter values.

#### 4.1.1. Model Comparison

Given the ADT data with different sample sizes, the relative errors (*RE*) of the parameter estimation in percentage are computed by:

(33) $$RE=\frac{Estimated.value-True.value}{True.value}\times 100.$$

To investigate the variance and the bias of the estimator, the relative square error (*RSE*) of parameters in percentage is also given by Equation (34):

(34) $$RSE=\frac{{\left(Estimated.value-True.value\right)}^{2}}{True.value^{2}}\times 100.$$

From the above settings, *M*_0_, *M*_1_ and *M*_2_ have 6, 4 and 5 parameters, respectively. Meanwhile, the absolute error (*AE*) of the candidate model *M_i_* (*i* = 0, 1, 2) to the real model *M_real_* is given by Equation (35) to quantitatively analyze the reliability evaluation results:

(35) $$AE\left(M_{i}|M_{real}\right)=\frac{1}{N_{t}}\sum_{j=1}^{N_{t}}\left[F_{T}\left(t_{j}|M_{real}\right)-F_{T}\left(t_{j}|M_{i}\right)\right],$$

where *F_T_*(*t*) is the CDF of the FPT at the normal stress level given by Equation (13), *t_j_* = 0.1, 1.1, …, 699.1, for hundreds of hours in this study, *N_t_* = 700. Herein, if *AE* > 0, it means that model *M_i_* overestimates the reliability evaluation results comparing with the true values, otherwise it underestimates.

**Figure 2:**
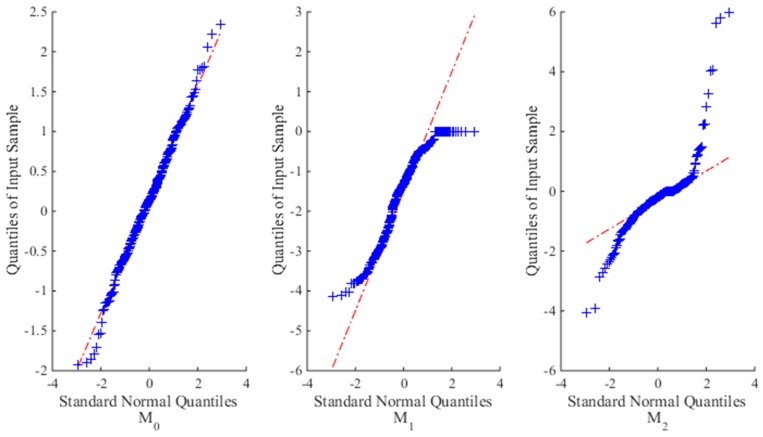
The Q-Q plots for three candidate models for the simulated SSADT data when *n* = 10.

Table 1 gives the estimates of unknown parameters with their *RE*s and *RSE*s, and *AE*s of reliability estimation for different models at different sample sizes. Figure 2 illustrates the fitting results of each model on the simulation data when *n* = 10. Clearly, from *l_max_* and Δ values at each sample size, *M*_0_ is the most suitable model, then is model *M*_2_, and the worst is model *M*_1_. The reason is that the time-scale transformation model *M*_2_ can capture the nonlinear property of the degradation process to some extent, but still perform worse than *M*_0_. As to *M*_1_, it tries to linearize the nonlinear degradation process, which leads to dreadful fitting results and poor parameter estimation compared with the true results.

From the *AE* values in Table 1, it is known that both model *M*_1_ and *M*_2_ overestimate the reliability evaluation results at normal stress levels. With the sample size increasing, the *AE*s for *M*_1_ and *M*_2_ become larger since that they are not the right models for ADT analysis and the level of error will be amplified when more data are available for model validation. Meanwhile, model *M*_0_ slightly underestimates the reliability evaluation results from *AE* indexes, and the accuracies are improved by several orders of magnitude when the sample size goes from five to 30. The results demonstrate that model *M*_0_ is the most applicable model for nonlinear ADT analysis and can provide accurate reliability and lifetime evaluation results.

**Figure 3:**
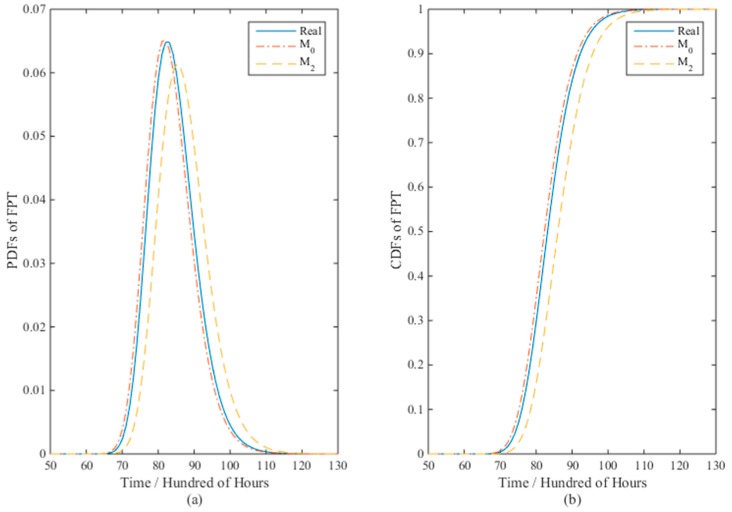
The (**a**) PDFs and (**b**) CDFs of the FPT for models *M*_0_ and *M*_2_ with the real values when *n* = 10.

Figure 3 presents the PDFs and CDFs of the FPT for models *M*_0_ and *M*_2_ with the real values when the sample size *n* = 10. It is clear that model *M*_0_ is closer to the real values than *M*_2_. The mean time to failure (MTTF) for *M*_0_ and *M*_2_ are 8340 and 8720 h, while the true value is 8430 h. The results verify the effectiveness of model *M*_0_ than *M*_2_ with respect to nonlinear ADT analysis.

#### 4.1.2. Sensitivity Analysis

In this section, sensitivity analysis is conducted to analyze the robustness of the general model *M*_0_ with different values of model parameters [*θ*, *γ*, *σ*^2^, *η*_0_ (i.e., *a*, *b*), *η*_1_] for the simulation example.

Here, we set the parameters’ real values to 90%, 95%, 100%, 105% and 110% as the five factor levels. If model *M*_0_ is robust, its relative error of reliability evaluation results at normal stress levels should be as small as possible when compared with the real model *M_real_*. Herein, we repeated the simulation procedure of SSADT data for *N_s_* = 100 times and *n* = 10 samples will be generated at each time point. Then, the relative error for model *M*_0_ (*RE* of *M*_0_) is given through Equation (36):

(36) $$RE\left(M_{0}\right)=\frac{1}{N_{s}}\sum_{k=1}^{N_{s}}\frac{1}{N_{t}}\sum_{j=1}^{N_{t}}\left|F_{T}^{k}\left(t_{j}|M_{0}\right)-F_{T}^{k}\left(t_{j}|M_{real}\right)\right|.$$

where $F_{T}^{k}(\cdot )$ is the CDF of the FPT at the normal stress level for the *k*th simulation.

Due to the extensive pairs of parameter combinations, say 5^6^ = 15,625 and *N_s_* = 100 repeated simulations, it will lead to heavy computational effort to compute the results. Thus, the orthogonal design of experiments is introduced to reduce the number of combinations, but still be able to find the sensitive parameters from the response (*RE* of *M*_0_) at each factor level [35]. The orthogonal array *L*_25_(5^6^) is selected with 25 overall tests rather than 5^6^.

The sensitivity analysis results are listed in Table 2, where the numbers from 1 to 5 refer to the first to fifth factor levels of the real values. For instance, in Test No. 1, the values are all 1, which means that 90% of all of the real values will be used to compute the corresponding response, which is 0.026812. The means of the responses for the five factor levels are listed at the bottom of Table 2, i.e., *MRj*, *j* = 1, 2, …, 5. The absolute biases are then calculated through *δ* = *max*(*MRj*) − *min*(*MRj*) which presents the influence of each parameter to the *RE* of *M*_0_. From Table 2, it is known that the sensitivity of the parameters is ranked as *θ* > *b* > *η*_1_ > *γ* > *σ*^2^ > *a* for the simulation study. Hence, special attention should be given to those parameters when using them for reliability and lifetime evaluation at normal stress levels. Furthermore, the absolute bias *δ* ranged from 0.01806 to 0.05734, indicating that model *M*_0_ is quite robust for lifetime and reliability evaluation.

**Figure 4:**
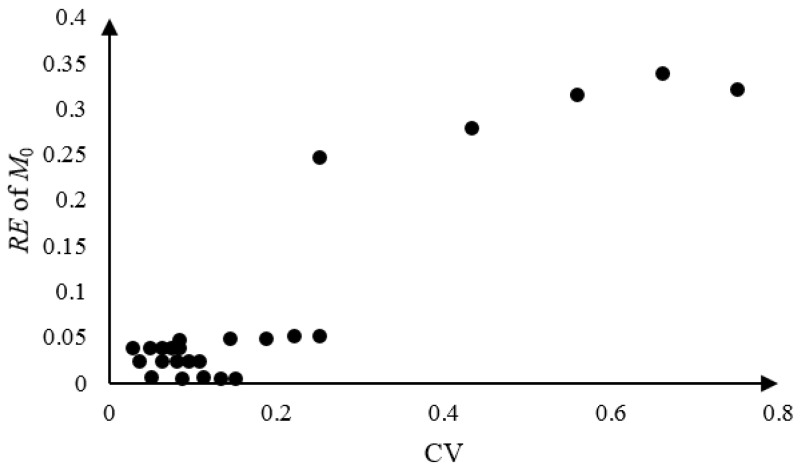
The correlation between CVs and *RE* of *M*_0_.

Since the parameters *a* and *b* contribute to the normal drift coefficient *μ*_0_, we may be interested in the performances of *M*_0_ on a wide range of the coefficients of variations (CVs). Five new factor levels are given to *a* and *b* with other parameters fixed, i.e., 20%, 60%, 100%, 140% and 180%. Twenty five tests were conducted with *N_s_* = 100 repeated simulations according to the values at the first three columns in Table 2. The results are shown in Figure 4. As we can see, the relative errors of *M*_0_ remain as significantly lower values than 0.05 with the CVs ranging 0~0.25. While the relative errors rise to the values around 0.3 with the CVs ranging 0.25~0.75, the robustness of *M*_0_ is still shown.

### 4.2. Real Applications

In the next two sections, two real ADT applications are used to further illustrate the advantages of *M*_0_ over *M*_1_ and *M*_2_ for both linear and nonlinear ADT analyses.

#### 4.2.1. LED Application

**Figure 5:**
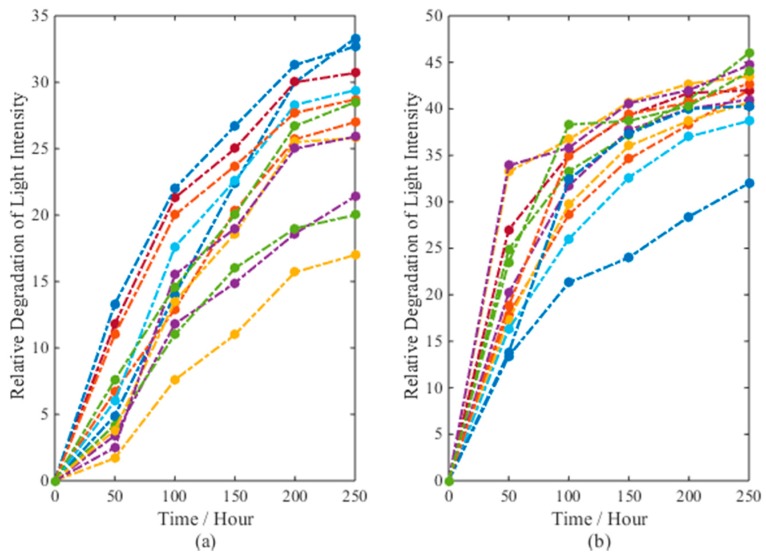
The degradation paths for twenty four LEDs under two electric current levels: (**a**) 35 mA and (**b**) 40 mA.

Light emitting diodes (LEDs) have the merit of longer lifetime, lower power consumption, and higher brightness than traditional light sources and, thus, are widely used in the area of lighting systems. The CSADT for LEDs is conducted under two electric current levels: 35 mA and 40 mA. The normal stress level is 25 mA. Twelve LEDs are tested at each stress level and the degradation data are recorded at 50, 100, 150, 200 and 250 h. The LED will be considered to have failed when the relative degradation value of the light intensity exceeds *ω* = 50. For the original data, refer to Chaluvadi [36] (Table 6.3). Figure 5 shows the degradation paths for the twenty-four tested samples under two stress levels.

In [36], a linear model is used to extrapolate the pseudo-failure time of each LED at two stress levels. Then, the inverse power law is used to determine the relation between the failure time data and the electric current stresses. This procedure is similar to model *M*_1_ in our approach. As we can see from Figure 5, the tested LEDs experience nonlinear degradation paths. Therefore, a linear model like *M*_1_ may not be appropriate for ADT analysis in this case. Thus, we use model *M*_0_ to fit the data and compare the results with model *M*_1_ and *M*_2_ to verify its effectiveness on nonlinear ADT analysis. The inverse power model is used as the acceleration model, i.e.,:

(37) $$\mu \left(I;\eta_{0},\eta_{1}\right)=\eta_{0}I^{\eta_{1}}.$$

**Figure 6:**
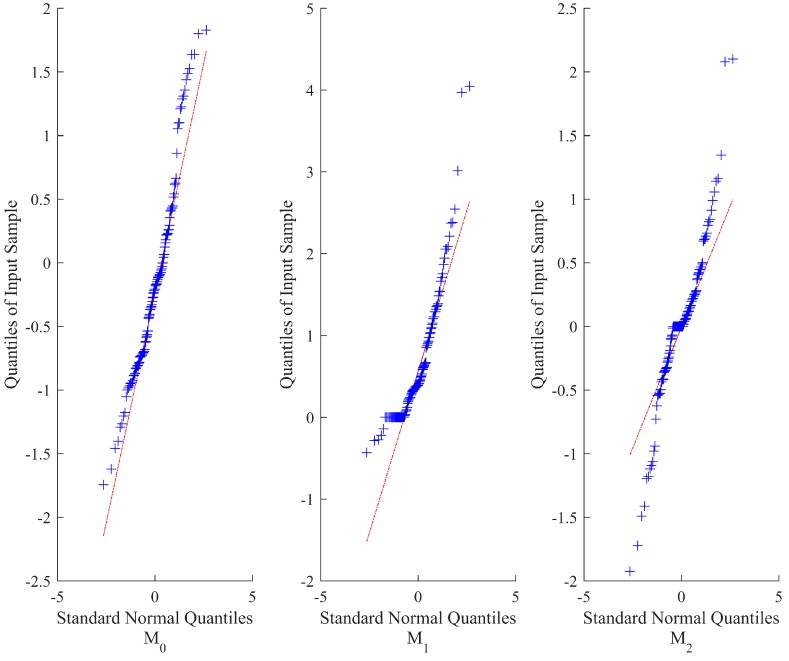
The Q-Q plots for three candidate models for the LED data.

Table 3 gives the estimates of unknown parameters according to the procedure in Section 3.1 and Section 3.2. It can be calculated that Pr(*μ*_0_ < 0) = 6.1870 × 10^−11^, which verifies that there is no danger of there being any negative drift coefficient. Figure 6 presents the fitting results. It is obvious that model *M*_0_ is the most applicable model with the maximum log-likelihood and lowest *AIC* value, which is the same as the fitting results. As with the linear model *M*_1_, the fitting results are worse than the other two models. Thus, the linear model in [36] is not appropriate for the LED application, while the performance of model *M*_2_ lies between model *M*_0_ and *M*_1_.

Additionally, as shown in Table 3, the *b* values are close to zero, which suggests a deterministic drift coefficient without the consideration of unit-to-unit variation. Hence, we consider this situation (*b* = 0) for all three models. The parameters are estimated through a one-stage MLE (see Table 4).

Compared with the *AIC* values in Table 3 and Table 4, it can be concluded that the models considering unit-to-unit variation (*b* ≠ 0) fit the LED data better than without consideration (*b* = 0), which indicates the presence of such variation. Additionally, among the three models, *M*_0_ should be selected for the LED ADT data analysis with the lowest *AIC* value.

**Figure 7:**
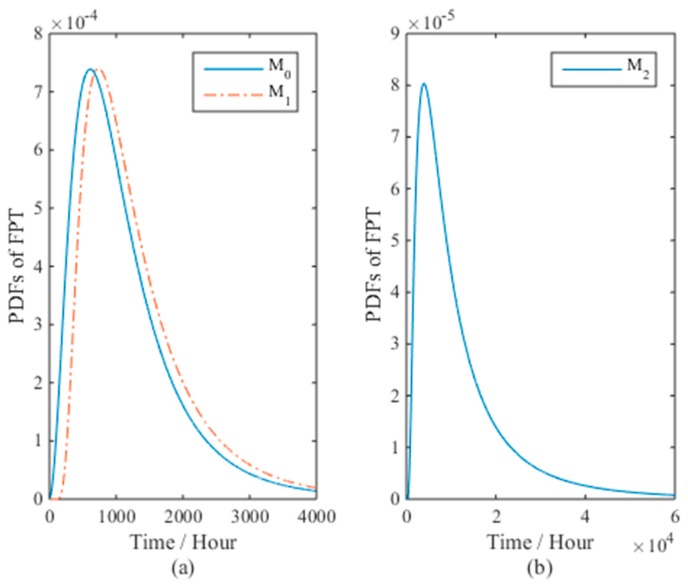
The PDFs of the FPT for different models in the LED case: (**a**) for *M*_0_ and *M*_1_, (**b**) for *M*_2_.

The PDFs of the FPT for different models (*b* ≠ 0) at normal stress levels are shown in Figure 7. The MTTF for *M*_0_, *M*_1_, and *M*_2_ are 1167.2, 1345.0, and 13,169.2 h, while the 95% confidence intervals are [202.1, 3459.1], [364.1, 3744.1], and [1660.1, 52,787.1] h, respectively. However, the estimated lifetime using the degradation-path model in Chaluvadi [36] (p. 103) is 1346 h. It is interesting to see that the intervals of *M*_0_ and *M*_1_ can capture this value to show the consistency of evaluation results under different models, while *M*_2_ computes significantly larger values, meaning that model *M*_2_ is unreliable. A reasonable explanation may be that the time-scale transformation is inapplicable for the nonlinear ADT analysis for LEDs. With respect to model *M*_1_, its lifetime evaluation results are closer to model *M*_0_, although it fits worse on the ADT data than model *M*_2_. In Tang, et al. [18], the time-scale transformation model *M*_2_ is used to analyze the same set of data and their 95% confidence interval is [1672, 53,466] h, which, however, is also not valid in the LED case, as discussed. For the proposed model *M*_0_, its evaluation results are reliable with the best nonlinear ADT data fitting.

#### 4.2.2. Resistor Application

**Figure 8:**
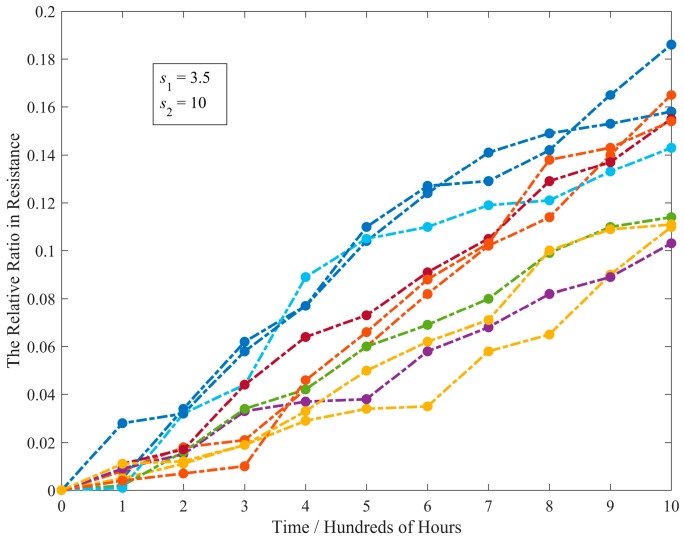
The degradation paths for ten resistors in CSADT (*s*_1_ = 3.5 and *s*_2_ = 10).

Carbon-film resistors are a fixed-form type of resistor and have superior characteristics to carbon composite resistors in terms of much closer tolerances, higher maximum ohmic values, and being used in high voltage and high temperature applications. Thus, the resistance of such a resistor is affected by the temperature (*s*_1_) and applied voltage (*s*_2_). Hence, in order to evaluate their lifetimes, the CSADT is conducted under nine constant stress levels with two acceleration variables, i.e., *s*_1_ = 3.5, 4.0 and 5.0 in hundred Kelvin, *s*_2_ = 10, 15 and 20 volts. The normal stress level is *s*_1_ = 3.2315 and *s*_2_ = 5. Ten resistors are tested at each stress level. For more details about the description of resistor data, readers are referred to Park and Padgett [25]. The original data is modified to ensure that the initial degradation values are equal to zero and the threshold *ω* is, therefore, set to be 0.2. The CSADT data at the first stress level is presented in Figure 8. It is clear that the degradation processes follow linear paths which are the same as the data in other stress levels.

Regarding the acceleration model with the unit-to-unit variation, the following model with two acceleration variables is used [25]:

(38) $$\mu \left(s_{1},s_{2};\eta_{0},\eta_{1},\eta_{2}\right)=\eta_{0}e^{\eta_{1}s_{1}}\cdot e^{\eta_{2}s_{2}}.$$

**Figure 9:**
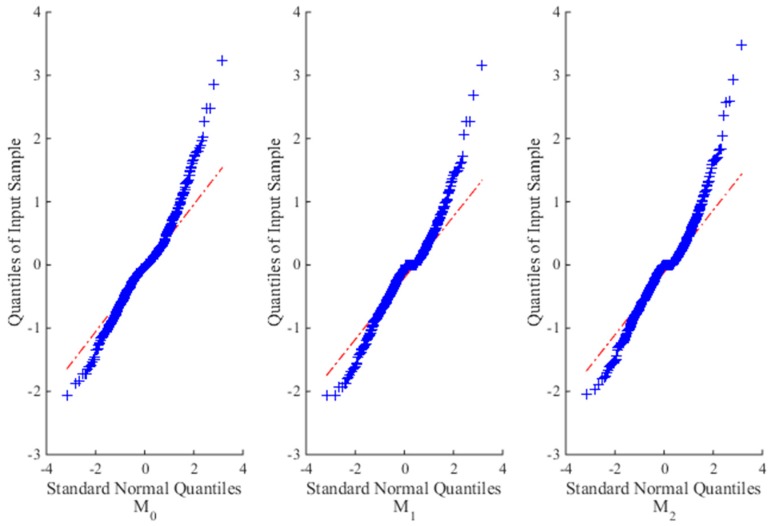
The Q-Q plots for three candidate models for the resistor data.

Table 5 presents the estimates of the unknown parameters for different models on resistor CSADT data. We also calculated that Pr(*μ*_0_ < 0) = 6.0094 × 10^−6^, which verifies that there is no danger of there being any negative drift coefficient. Intuitively, compared with *M*_1_ and *M*_2_, model *M*_0_ displays the best fit with the maximum log-likelihood value and minimum *AIC* value. However, according to the Δ values, there is substantial support for model *M*_2_ and considerably less support for model *M*_1_. The fitting results for the different models are shown in Figure 9, which imply that the performances of those models are similar in the resistor case since that the degradation paths are approximately linear.

Herein, we also checked the models without the consideration of unit-to-unit variation. The results are listed in Table 6. Compared with the results in Table 5, it can be seen that models with the normal drift coefficients (*b* ≠ 0) have lower *AIC* values, which means that the unit-to-unit variation should be considered. It is also interesting to see that, from Table 6, *M*_1_ is the best model with a deterministic drift coefficient, while supports are given to the other two models.

We may also be interested in whether *M*_0_ can be simplified into *M*_2_ with the assumption (*θ* = *γ*) since *M*_2_ has substantial support. Hence, the likelihood ratio (LR) test is implemented with the log-likelihood values in Table 5. The resulting LR statistic is 3.57 (<$\chi_{1,0.05}^{2}$ = 3.84). Thus, we accept the assumption and choose *M*_2_.

**Figure 10:**
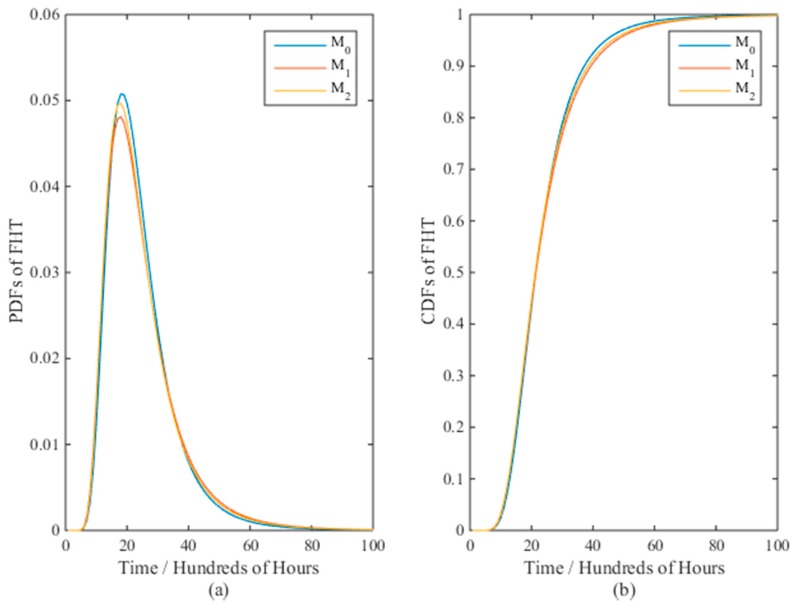
The (**a**) PDFs and (**b**) CDFs of the FPT for three candidate models in the resistor case.

The PDFs and CDFs of the FPT for different models are given in Figure 10a,b, which are almost identical with the MTTF at around 2400 h, and the PDF of the FPT for model *M*_0_ is slightly sharper than that of *M*_1_ and *M*_2_.

Comparing Section 4.1 with Section 4.2, it can be concluded that the proposed method can be effectively used for ADT analysis for both linear and nonlinear scenarios with single and multiple acceleration variables.

## 5. Conclusions

This paper has proposed a general ADT model based on the Wiener process and provided statistical analysis methods for unknown parameter estimation. The general ADT model is suitable for both linear and nonlinear ADT analyses, with single and multiple acceleration variables, and considers the unit-to-unit variation among the tested samples and temporal variation of the degradation processes simultaneously. The simulation example demonstrates that the general model is robust with respect to ADT analysis and its reliability evaluation results become more accurate when the sample size increases. Furthermore, the LED and resistor cases have verified its effectiveness and superiority to real engineering applications over the commonly used linear and time-scale transformation Wiener process models, which not only fit well with the degradation data at all accelerated stress levels, but also computes reliable lifetime and reliability evaluation results.

This study focused on the modeling procedure for ADT data based on the Wiener process. However, other methods, e.g., the Gamma or inverse Gaussian processes, are worth further research when the degradation paths are monotonic.

## Figures and Tables

**Table 1:** Simulation example: parameter estimates with *RE*s and *RSE*s in percentage (in parentheses), and *AE*s of reliability estimation for three candidate models under three sample sizes.

*M_i_*	*n*	*θ*	*γ*	*σ*^2^	*η*_0_		*η*_1_	*l_max_*	*n_p_*	*AIC*	Δ	*AE*
					*a*	*b*						
*M*_0_	5	1.491	0.273	0.0177	21.62	4.33	–1502	317	6	–621	0	–2.9×10^−3^
		(−0.57, 3.3 × 10^−3^)	(−32, 10)	(77, 59)	(8.1, 0.65)	(−13, 1.8)	(0.13, 1.7 × 10^−4^)					
	10	1.502	0.381	0.0097	18.65	4.42	–1477	635		–1259	0	–1.2 × 10^−3^
		(0.14, 2.0 × 10^−4^)	(−4.7, 0.22)	(−3.1, 0.096)	(−6.7, 0.45)	(−12, 1.3)	(−1.5, 0.023)					
	30	1.501	0.352	0.0114	20.90	5.18	–1515	1898		–3784	0	2.7 × 10^−4^
		(0.055, 3.0 × 10^−5^)	(−12, 1.4)	(14, 2.1)	(4.5, 0.20)	(3.6, 0.13)	(1.0, 0.011)					
*M*_1_	5	1 (fixed)	1 (fixed)	0.0607	8.5 × 10^3^	7.9 × 10^5^	–3045	−2.7	4	13.4	635	0.343
		(−33, 11)	(150, 225)	(507, 2.6 × 10^3^)	(4.2 × 10^4^, 1.8 × 10^7^)	(1.6 × 10^7^, 2.5 × 10^12^)	(103, 106)					
	10	1 (fixed)	1 (fixed)	0.0579	8.2 × 10^3^	8.3 × 10^5^	–3049	1.7		4.6	1263	0.365
		(−33, 11)	(150, 225)	(479, 2.3 × 10^3^)	(4.1 × 10^4^, 1.7 × 10^7^)	(1.7 × 10^7^, 2.8 × 10^12^)	(103, 107)					
	30	1 (fixed)	1(fixed)	0.0578	9.2 × 10^3^	1.1 × 10^6^	–3087	5.5		–3.0	3781	0.374
		(−33, 11)	(150, 225)	(478, 2.3 × 10^3^)	(4.6 × 10^4^, 2.1 × 10^7^)	(2.2 × 10^7^, 4.6 × 10^12^)	(106, 112)					
*M*_2_	5	1.461	=*θ*	7.29 × 10^−4^	29.55	8.27	–1577	217	5	–425	197	3.2 × 10^−3^
		(−2.6, 0.068)	(265, 704)	(−93, 86)	(48, 23)	(65, 43)	(5.1, 0.26)					
	10	1.476	=*θ*	4.79 × 10^−4^	24.54	7.64	–1544	490		–971	288	4.2 × 10^−3^
		(−1.6, 0.026)	(269, 724)	(−95, 91)	(23, 5.2)	(53, 28)	(2.9, 0.087)					
	30	1.477	=*θ*	5.87 × 10^−4^	26.96	8.58	–1577	1380		–2750	1034	5.3 × 10^−3^
		(−1.6, 0.025)	(269, 724)	(−94, 89)	(35, 12)	(72, 51)	(5.2, 0.27)					

**Table 2:** Sensitivity analysis of *M*_0_ with five levels of parameters through the orthogonal array *L*_25_(5^6^) and Taguchi analysis.

Test No.	*θ*	*γ*	*σ*^2^	*η*_0_		*η*_1_	*RE* of *M*_0_
				*a*	*b*		
1	1	1	1	1	1	1	0.026812
2	1	2	2	2	2	2	0.049430
3	1	3	3	3	3	3	0.076352
4	1	4	4	4	4	4	0.107904
5	1	5	5	5	5	5	0.149784
6	2	1	2	3	4	5	0.101002
7	2	2	3	4	5	1	0.017453
8	2	3	4	5	1	2	0.005818
9	2	4	5	1	2	3	0.042759
10	2	5	1	2	3	4	0.068059
11	3	1	3	5	2	4	0.014726
12	3	2	4	1	3	5	0.060681
13	3	3	5	2	4	1	0.032238
14	3	4	1	3	5	2	0.018415
15	3	5	2	4	1	3	0.006743
16	4	1	4	2	5	3	0.019738
17	4	2	5	3	1	4	0.008050
18	4	3	1	4	2	5	0.010650
19	4	4	2	5	3	1	0.053845
20	4	5	3	1	4	2	0.031300
21	5	1	5	4	3	2	0.053440
22	5	2	1	5	4	3	0.044617
23	5	3	2	1	5	4	0.021416
24	5	4	3	2	1	5	0.009040
25	5	5	4	3	2	1	0.061280
*MR*1	0.08206	0.04314	0.03371	0.03659	0.01129	0.03833	*T* = 1.09155
*MR*2	0.04702	0.03605	0.04649	0.03570	0.03577	0.06138	
*MR*3	0.02656	0.02929	0.02977	0.05302	0.06248	0.03804	
*MR*4	0.02472	0.04639	0.05108	0.03924	0.06341	0.04403	
*MR*5	0.03796	0.06343	0.05725	0.05376	0.04536	0.06623	
*δ*	0.05734	0.03414	0.02748	0.01806	0.05212	0.03455	
Rank	1	4	5	6	2	3	

**Table 3:** LED application: parameter estimates for three candidate models with random drift coefficients (*b* ≠ 0).

Model	*θ*	*γ*	*σ*^2^	*η*_0_		*η*_1_	*l_max_*	*n_p_*	*AIC*	Δ
				*a*	*b*					
*M*_0_	0.442	0.117	73.784	0.273	0.0018	0.677	–310	6	633	0
*M*_1_	1 (fixed)	1 (fixed)	0.761	1.69 × 10^−6^	5.62 × 10^−14^	3.112	–389	4	785	152
*M*_2_	0.450	=***θ***	5.840	3.52 × 10^−5^	2.43 × 10^−11^	3.112	–317	5	644	11

**Table 4:** LED application: parameter estimates for three candidate models with deterministic drift coefficients (*b* = 0).

Model	*θ*	*γ*	*σ*^2^	*η*_0_ = *a*	*η*_1_	*l*_max_	*n_p_*	*AIC*	Δ
*M*_0_	0.448	0.171	45.429	0.012	1.179	–314.6634	5	639	0
*M*_1_	1 (fixed)	1 (fixed)	0.776	8.67 × 10^−7^	3.297	–389.7358	3	785	146
*M*_2_	0.4477	=***θ***	6.238	1.83 × 10^−5^	3.297	–319.9119	4	648	9

**Table 5:** Resistor application: parameter estimates for three candidate models with random drift coefficients (*b* ≠ 0).

*Model*	*θ*	*γ*	*σ*^2^	*η*_0_		*η*_1_	*η*_2_	*l_max_*	*n_p_*	*AIC*	Δ
				*a*	*b*						
*M*_0_	1.076	0.918	3.02 × 10^−4^	8.96 × 10^−4^	4.19 × 10^−8^	0.462	0.108	1698	7	–3383	0
*M*_1_	1 (fixed)	1 (fixed)	2.59 × 10^−4^	0.0012	7.02 × 10^−8^	0.440	0.102	1694	5	–3378	4.8
*M*_2_	1.046	=***θ***	2.35 × 10^−4^	0.0010	5.12 × 10^−8^	0.451	0.106	1697	6	–3381	1.6

**Table 6:** Resistor application: parameter estimates for three candidate models with deterministic drift coefficients (*b* = 0).

Model	*θ*	*γ*	*σ*^2^	*η*_0_ = *a*	*η*_1_	*η*_2_	*l*_max_	*n_p_*	*AIC*	Δ
*M*_0_	1.020	0.9498	3.33 × 10^−4^	0.0011	0.445	0.104	1647	6	–3282	2.7
*M*_1_	1 (fixed)	1 (fixed)	3.02 × 10^−4^	0.0012	0.439	0.103	1646	4	–3284	0
*M*_2_	1.008	=***θ***	2.97 × 10^−4^	0.0011	0.442	0.103	1646	5	–3283	1.8

## References

[1] Elsayed E.A., Chen A.C.K. Recent research and current issues in accelerated testing. Proceedings of the 1998 IEEE International Conference on Systems, Man, and Cybernetics.

[2] Meeker W.Q., Escobar L.A. (1998). Statistical Methods for Reliability Data.

[3] Nelson W.B. (1990). Accelerated Testing: Statistical Models, Test Plans, and Data Analysis.

[4] Thomas E.V., Bloom I., Christophersen J.P., Battaglia V.S. (2008). Statistical methodology for predicting the life of lithium-ion cells via accelerated degradation testing. J. Power Sources.

[5] Bae S.J., Kim S.-J., Park J.I., Lee J.-H., Cho H., Park J.-Y. (2010). Lifetime prediction through accelerated degradation testing of membrane electrode assemblies in direct methanol fuel cells. Int. J. Hydrogen Energy.

[6] Wang F.-K., Lu Y.-C. (2014). Useful lifetime analysis for high-power white LEDs. Microelectron. Reliab..

[7] Wang F.-K., Chu T.-P. (2012). Lifetime predictions of LED-based light bars by accelerated degradation test. Microelectron. Reliab..

[8] Meeker W.Q., Escobar L.A., Lu C.J. (1998). Accelerated degradation tests: Modeling and analysis. Technometrics.

[9] Park J.I., Bae S.J. (2010). Direct prediction methods on lifetime distribution of organic light-emitting diodes from accelerated degradation tests. IEEE Trans. Reliab..

[10] Ling M.H., Tsui K.L., Balakrishnan N. (2015). Accelerated degradation analysis for the quality of a system Based on the gamma process. IEEE Trans. Reliab..

[11] Whitmore G.A., Schenkelberg F. (1997). Modelling accelerated degradation data using wiener diffusion with A time scale transformation. Lifetime Data Anal..

[12] Ye Z.S., Chen L.P., Tang L.C., Xie M. (2014). Accelerated degradation test planning using the inverse gaussian process. IEEE Trans. Reliab..

[13] Escobar L.A., Meeker W.Q. (2006). A review of accelerated test models. Stat. Sci..

[14] Park C., Padgett W.J. (2005). Accelerated degradation models for failure based on geometric Brownian motion and gamma processes. Lifetime Data Anal..

[15] Pan Z.Q., Balakrishnan N. (2010). Multiple-steps step-stress accelerated degradation modeling based on wiener and gamma processes. Commun. Stat. Simul. Comput..

[16] Wang L.Z., Pan R., Li X.Y., Jiang T.M. (2013). A Bayesian reliability evaluation method with integrated accelerated degradation testing and field information. Reliab. Eng. Syst. Saf..

[17] Liao H.T., Elsayed E.A. (2006). Reliability inference for field conditions from accelerated degradation testing. Nav. Res. Logist..

[18] Tang S.J., Guo X.S., Yu C.Q., Xue H.J., Zhou Z.J. (2014). Accelerated degradation tests modeling based on the nonlinear wiener process with random effects. Math. Probl. Eng..

[19] Wang X.L., Jiang P., Guo B., Cheng Z.J. (2014). Real-time reliability evaluation with a general wiener process-based degradation model. Qual. Reliab. Eng. Int..

[20] Wang X.L., Balakrishnan N., Guo B. (2014). Residual life estimation based on a generalized Wiener degradation process. Reliab. Eng. Syst. Saf..

[21] Liu L., Li X.-Y., Jiang T.-M. Nonlinear accelerated degradation analysis based on the general wiener process. Proceedings of the 25th European Safety and Reliability Conference (ESREL 2015).

[22] Peng C.Y., Tseng S.T. (2009). Mis-specification analysis of linear degradation models. IEEE Trans. Reliab..

[23] Wang X. (2010). Wiener processes with random effects for degradation data. J. Multivar. Anal..

[24] Si X.S., Wang W.B., Hu C.H., Zhou D.H., Pecht M.G. (2012). Remaining useful life estimation based on a nonlinear diffusion degradation process. IEEE Trans. Reliab..

[25] Park C., Padgett W.J. (2006). Stochastic degradation models with several accelerating variables. IEEE Trans. Reliab..

[26] Li X., Jiang T., Sun F., Ma J. Constant stress ADT for superluminescent diode and parameter sensitivity analysis. Proceedings of the 8th International Conference on Reliability, Maintainability and Safety.

[27] Lim H., Yum B.-J. (2011). Optimal design of accelerated degradation tests based on Wiener process models. J. Appl. Stat..

[28] Si X.-S., Wang W., Hu C.-H., Chen M.-Y., Zhou D.-H. (2013). A Wiener-process-based degradation model with a recursive filter algorithm for remaining useful life estimation. Mech. Syst. Signal Process..

[29] Chhikara R.S., Folks J.L. (1988). The Inverse Gaussian Distribution: Theory, Methodology, and Applications.

[30] Ye Z.-S., Xie M. (2015). Stochastic modelling and analysis of degradation for highly reliable products. Appl. Stoch. Model. Bus..

[31] Tang L.C., Yang G., Xie M. Planning of step-stress accelerated degradation test. Proceedings of the 2004 Annual Reliability and Maintainability Symposium.

[32] Lagarias J.C., Reeds J.A., Wright M.H., Wright P.E. (1998). Convergence properties of the Nelder-Mead simplex method in low dimensions. SIAM J. Optim..

[33] Van Noortwijk J.M. (2009). A survey of the application of gamma processes in maintenance. Reliab. Eng. Syst. Saf..

[34] Burnham K.P., Anderson D.R. (2004). Multimodel inference understanding AIC and BIC in model selection. Sociol. Method Res..

[35] Taguchi G., Yokoyama Y. (1993). Taguchi Methods: Design of Experiments.

[36] Chaluvadi V. (2008). Accelerated Life Testing of Electronic Revenue Meters. Master Thesis.

